# Arcuate Fasciculus Abnormalities and Their Relationship with Psychotic Symptoms in Schizophrenia

**DOI:** 10.1371/journal.pone.0029315

**Published:** 2012-01-05

**Authors:** Muhammad Farid Abdul-Rahman, Anqi Qiu, Puay San Woon, Carissa Kuswanto, Simon L. Collinson, Kang Sim

**Affiliations:** 1 Division of Bioengineering, National University of Singapore, Singapore; 2 Clinical Imaging Research Center, National University of Singapore, Singapore; 3 Singapore Institute for Clinical Sciences, Agency for Science, Technology and Research, Singapore; 4 Research Division, Institute of Mental Health, Singapore; 5 Department of Psychology, Institute of Mental Health, Singapore; 6 Department of General Psychiatry, National University of Singapore, Singapore; Institute of Automation, Chinese Academy of Sciences, China

## Abstract

Disruption of fronto-temporal connections involving the arcuate fasciculus (AF) may underlie language processing anomalies and psychotic features such as auditory hallucinations in schizophrenia. No study to date has specifically investigated abnormalities of white matter integrity at particular loci along the AF as well as its regional lateralization in schizophrenia. We examined white matter changes (fractional anisotropy (FA), axial diffusivity (AD), asymmetry indices) along the whole extent of the AF and their relationship with psychotic symptoms in 32 males with schizophrenia and 44 healthy males. Large deformation diffeomorphic metric mapping and Fiber Assignment Continuous Tracking were employed to characterize FA and AD along the geometric curve of the AF. Our results showed that patients with schizophrenia had lower FA in the frontal aspects of the left AF compared with healthy controls. Greater left FA and AD lateralization in the temporal segment of AF were associated with more severe positive psychotic symptoms such as delusions and hallucinations in patients with schizophrenia. Disruption of white matter integrity of the left frontal AF and accentuation of normal left greater than right asymmetry of FA/AD in the temporal AF further support the notion of aberrant fronto-temporal connectivity in schizophrenia. AF pathology can affect corollary discharge of neural signals from frontal speech/motor initiation areas to suppress activity of auditory cortex that may influence psychotic phenomena such as auditory hallucinations and facilitate elaboration of delusional content.

## Introduction

Extant data from postmortem [1], neuroimaging [2], [3], [4] and genetic studies [5], [6] have pointed towards abnormalities of the white matter in schizophrenia. With respect to neuroimaging of white matter structures in schizophrenia, voxel-based morphometry studies found white matter reductions in frontal, temporal, parietal and occipital lobes [7]. Diffusion tensor imaging (DTI) studies adopting voxel-based analyses and tractography techniques have allowed detailed examination of the organization of these white matter regions. These studies suggested that one of the white matter tracts consistently implicated in schizophrenia is the arcuate fasciculus (AF), an associative fiber tract that connects the frontal cortex with the parietal and temporal cortices. In schizophrenia, reductions of FA either bilaterally [8], [9] or especially on the left side of the AF have been reported [10], [11]. Of note, the AF is a neural pathway that connects language related regions such as the posterior segment of the temporo-parietal junction (Wernicke's area) with the frontal cortex and may underlie language processing anomalies found in schizophrenia [12], [13], [14]. The same pathway is involved in auditory and speech perception during covert and overt speech [15], [16], [17] and disruption of such fronto-temporal connections is thought to contribute to psychotic experiences such as auditory hallucinations [18]. Functional imaging studies in schizophrenia with auditory hallucinations have revealed involvement of brain regions connected through the AF [19], [20]. Abnormalities of the AF in schizophrenia have been associated with severity of auditory hallucinations and duration of illness [11], [21]. Since the AF connects distinct functional regions, better appreciation of the specific disruptions of white matter integrity along the course of the AF fronto-temporally may thus potentially help to understand the course of disease progression and provide a neural marker or correlate of psychopathology.

In addition, few studies have examined white matter asymmetries along the AF and other associative white matter fibers despite suggestions of asymmetric abnormalities and a possible relationship with clinical features [22], [23]. Chance et al. [24] examined gross cerebral asymmetries from structural MRI images of patients with schizophrenia and control subjects and found disturbance in the normal differential tissue distribution within the hemispheres along the anterior-posterior axis (also known as brain torque). More recent studies have suggested that global asymmetries may be more subtle and local than previously thought and affect only specific lobes and/or substructures [25]. Previous studies have also found that cortical structures, such as Heschl's gyrus and planum temporale, showed reduction or reversal of the normal left more than right asymmetry [26] which have been reported to be correlated with severity of symptoms in schizophrenia [27], [28] thus prompting the question of whether similar asymmetries occur in their adjacent white matter tract of the AF and the correlation with clinical symptoms are limited to the temporal segment of the AF.

In this study, we aimed to determine whether abnormalities of the white matter integrity and asymmetry of the AF and their relation with psychotic symptoms in schizophrenia only occur in its specific loci that are most functionally relevant. We employed tract-based analysis instead of traditional voxel-based analysis for several reasons. First, tract-based analysis allows characterization of the nature of white matter integrity and asymmetry indexes along the AF tract from the temporal lobe to the frontal lobe. Second, with tract-based analysis, there is no necessity for spatial smoothing of the 3D image volume which may render localization of abnormalities challenging to interpret in terms of the white matter tracts. Third, previous studies have shown that compared to voxel-based analysis, tract-based analysis increases sensitivity to detect changes due to its explicit incorporation of geometrical models of white matter tracts [29], [30], [31], [32], [33]. Using tract-based analysis, we examined the AF tract that is the most prominent bundle within the superior longitudinal fasciculus and rostrally extends to the precentral gyrus. This precise AF anatomy observed in recent neuroimaging studies [34], [35], [36], improves upon earlier notions of the AF fiber only passing through the temporal cortex and the pars opercularis and we sought to understand its specific role in schizophrenia. Based on previous findings, we hypothesized that firstly, white matter integrity of the AF is compromised at specific segments (frontal versus temporal lobes) of the AF in schizophrenia and secondly, the AF regional lateralization would be correlated with positive symptoms in schizophrenia.

## Methods

### Subjects

Thirty two right-handed males with schizophrenia and forty four handedness-matched healthy males were recruited from the Institute of Mental Health, Singapore and the community respectively for this study. The study was approved by the Institutional Review Boards of the Institute of Mental Health, Singapore, as well as that of the National Neuroscience Institute, Singapore. All schizophrenia subjects were recruited at their stable stage and thus were capable of making consent. All subjects gave their written informed consent following a complete description of the study. Socio-demographic and clinical information for the two groups of subjects are given in Table 1.

**Table 1:** Demographic and clinical characteristics of the sample.

Demographic/clinical feature	CON(N = 44)	SCZ(N = 32)	Test statistic	P value
Age (SD), years	30.39 (7.97)	38.28 (9.46)	*t_74_* = 3.938	<0.001
Years of Education (SD), years	14.1 (1.66)	11.5 (2.16)	*t_74_* = −5.903	<0.001
Mean Illness Duration (SD), years	-	13.8 (8.77)	-	-
PANSS positive symptom	-	10.66 (3.62)	-	-
PANSS negative symptom	-	8.88(2.98)	-	-
PANSS general psychopathology	-	20.4 (4.51)	-	-
PANSS positive symptom subscore- delusions	-	2.16 (1.25)	-	-
PANSS positive symptom subscore- hallucinatory behavior	-	1.91 (1.17)	-	-

Abbreviations: CON, control subjects; SCZ, patients with schizophrenia; SD, standard deviation.

All diagnoses were made by a psychiatrist using information obtained from the clinical history, mental status examination, existing medical records, interviews with significant others as well as the administration of the structured clinical interview for DSM-IV disorders-Patient Version (SCID-P) [37]. The patients were maintained on a stable dose of antipsychotic medications for at least two weeks prior to recruitment and did not have their medications withdrawn for the purpose of the study. Twenty one patients received second generation antipsychotics, eleven patients were prescribed first generation antipsychotics. The mean (SD) antipsychotic dose was 210.05 (120.16) daily chlorpromazine equivalents in milligrams. No subject met DSM-IV criteria for alcohol or other substance abuse within the preceding three months. The Positive and Negative Syndrome Scale (PANSS) [38] was used to assess the nature and severity of psychopathology. Both scales were administered by a psychiatrist to all the participants. The healthy controls were screened using the SCID-NP [39] to be free of any Axis I psychiatric disorder. None of the subjects had a history of major neurological, medical illnesses, substance abuse or psychotropic medication use.

### DTI acquisition and preprocessing

Single-shot echo-planar DTI (TR = 3725 ms; TE = 56 ms; flip angle = 90°) was acquired using a 3-Tesla whole body Philips scanner with a SENSE head coil. 42 axial slices with 3.0 mm thickness were acquired parallel to the anterior–posterior commissure line; the imaging matrix was 112×109 with a field of view of 230 mm×230 mm, which was zero-filled to 256×256. 15 diffusion weighted images (DWIs) with b = 800 sec/mm^2^ and 1 baseline with b = 0 sec/mm^2^ were obtained.

Within each subject, DWIs were first corrected for motion and eddy current distortions using affine transformation to the image without diffusion weighting. These DWIs were then registered to Mori's single-subject DTI atlas (resolution: 1×1×1 mm^3^, http://www.mristudio.org) [40] via an extensively validated approach involving affine and LDDMM nonlinear transformation between the images without diffusion weighting and FA images [41]. For each individual subject, the tensor, FA and AD values were recomputed using the DWIs aligned to Mori's DTI atlas via the diffeomorphic transformation. In addition, the mean DWIs were also constructed by averaging the corresponding DWIs across healthy controls. The tensor and FA value were computed from the mean DWIs in Mori's atlas space to represent the white matter anatomy in this study.

### Arcuate Fasiculus Delineation

**Figure 1:**
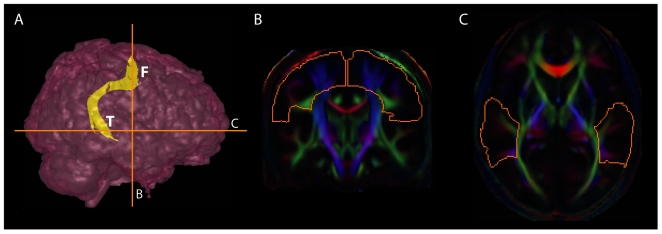
Delineation of the arcuate fasciculus (AF). Panel (A) illustrates the 3D lateral view of the brain in pink and the AF bundle is colored in yellow. The AF extends from the temporal lobe (labeled as “T”) to the frontal lobe (labeled as “F”). Panels (B, C) show the coronal and axial slices of the mean DTI color map, where the ROI boundaries in orange are shown for the AF delineation.

The AF in each hemisphere was reconstructed from the mean DTI using the Fiber Assignment Continuous Tracking (FACT) method [42] with a FA threshold of 0.15 and an angle threshold of 60°. The FACT tracking was first performed from all pixels inside the brain. Then, the AF was extracted using a two-ROI approach, that is, all tracts penetrating these endpoint ROIs were assigned to this bundle for further analysis. The two endpoint ROIs were manually drawn on the mean color map in Mori's DTI atlas space guided by the previously validated protocol [43]. The first ROI was placed on the coronal plane cutting the posterior limb of the internal capsule to encompass the intense green tract lateral to the posterior limb of the internal capsule and the branches emanating from this area (Figure 1B, coronal slice #120). Then, the second endpoint ROI was drawn on the axial plane lateral to the sagittal stratum (Figure 1C, axial slice #113). Since these ROIs were drawn in the averaged DTI aligned to the Mori's DTI atlas space, the tracts in the AF bundle are reproducible given the ROIs' coordinates.

Mean curves were computed to represent the orientation and shape of the left and right AF bundles using the LDDMM curve mapping [44], [45] based on all fiber tracts of the AF. As suggested in [46], we reparametrized each mean curve by its arc length and discretized it into equally spaced 50 points, which gave the anatomical correspondence between the left and right AF bundles in a point-to-point manner. Finally, we characterized the FA, AD values as functions of the location along the mean curve, which facilitated the detection of AF abnormalities in schizophrenia.

### Statistical Analysis

Demographic variables between patients with schizophrenia and control subjects were compared using two sample Student's *t*-test for continuous variables and chi-square test for categorical variables.

### Group Comparisons

In ROI-based analysis, we computed FA and AD values averaged over the AF bundle for individuals. Linear regression analyses with a main factor of diagnostic group (control vs schizophrenia) and covariates of age, years of education, and illness duration were performed on the mean FA and AD values to elucidate any abnormalities of the AF in schizophrenia compared with controls. In tract-based analysis, the FA and AD values along the tract were first smoothed using a moving window smoothing approach with window size of 5. Then, the same linear regression model was performed on FA and AD at every location of the AF mean curve. The statistical results were visualized at an overall significance level of 0.05 after Bonferroni correction. The uncorrected p-value was determined as 0.05/50*5 = 0.001, where 50 indicated the number of points on the AF and 5 was considered as the level of smoothness.

### Asymmetry

We computed lateralization index (LI) according to 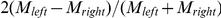, where 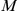 denotes FA or AD, to study the AF asymmetry [47]. The larger and more positive the index, the greater is the left anisotropy or diffusivity relative to the right side. We referred it as “leftward asymmetry”. Conversely, the greater the negativity, the greater is the right anisotropy or diffusivity relative to the left side (rightward asymmetry). In ROI-based analysis, comparison of LI away from 0 to identify asymmetry within healthy controls or patients with schizophrenia as well as asymmetric differences between the two groups in terms of FA or AD were examined using the same linear regression model described above. In tract-based analysis, the same comparison was applied to FA or AD at every location of the AF mean curve. The statistical results were visualized at an overall significance level of 0.05 after Bonferroni correction.

### Correlation with Schizophrenic Symptoms

To investigate the relation of the AF white matter integrity and its asymmetry with symptoms of schizophrenia characterized by PANSS subscores, partial correlation analysis was performed when controlling for the effects of age, years of education, and illness duration.

## Results

### Demographic and Clinical Characteristics

Compared to healthy controls, patients with schizophrenia were older (p<0.001) and had fewer years of education (p<0.001). The average duration of illness in patients with schizophrenia was 11.5 years.

### AF abnormalities in schizophrenia

ROI-based analysis only showed a trend of mean FA reduction in both the left and right AF but not mean AD in schizophrenia patients when compared to healthy controls (**Table 2**).

**Table 2:** White matter measures of the arcuate fasciculus (AF) using diffusion tensor imaging (DTI).

DTI measures of AF	CONMean (SD)	SCZMean (SD)	t-value	p-value
Left Mean FA	0.429 (0.028)	0.416 (0.031)	−1.772	0.081
Right Mean FA	0.398 (0.031)	0.381 (0.028)	−1.678	0.098
Left Mean AD (×10^−3^ mm^2^/s)	1.148 (0.036)	1.134 (0.039)	−0.866	0.389
Right Mean AD (×10^−3^ mm^2^/s)	1.122 (0.037)	1.134 (0.047)	−0.366	0.715
Mean LI-FA	0.077 (0.090)	0.087 (0.0919)	0.023	0.982
Mean LI-AD	0.023 (0.034)	−2.65e-04 (0.044)	−0.381	0.704

Abbreviations: AD, axial diffusivity; AF, arcuate fasciculus; DTI, diffusion tensor imaging; FA, fractional anisotropy; LI, lateralization indices.

**Figure 2:**
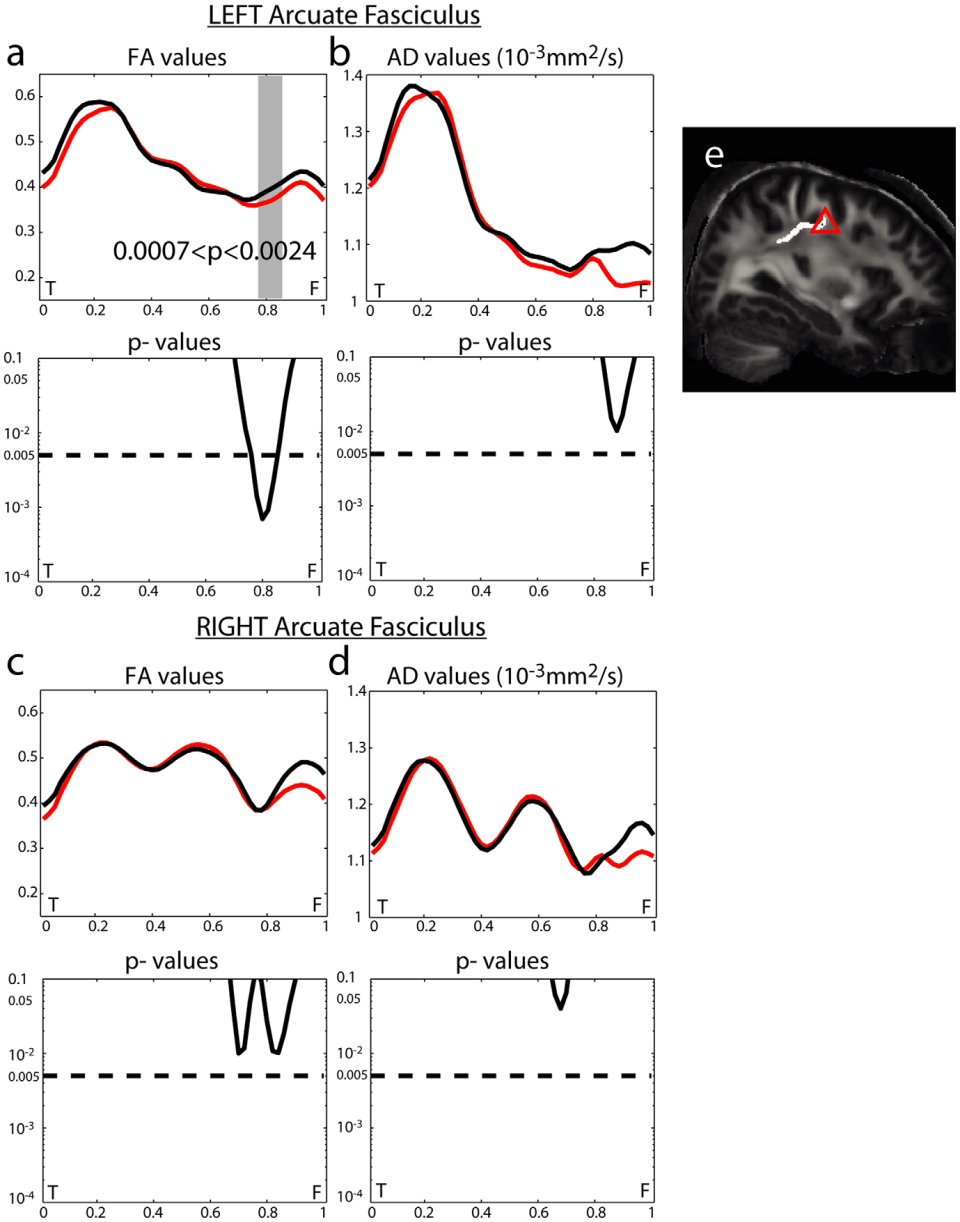
Tract-based analysis of DTI measures along the arcuate fasciculus (AF). On the top row of columns (A–D) the black line denotes the profile averaged over healthy controls, while the red line denotes the profile averaged over patients with schizophrenia. The region in gray denotes the anatomical location with significant group difference at p<0.05 after Bonferroni correction as marked by the dashed line on the bottom row of columns (A–D). The x-axis coordinates on panels (A–D) are normalized by the arc length of the AF. Panel (E) shows the triangle corresponding to the AF loci with a significant difference between healthy controls and patients with schizophrenia.

As illustrated in **Figure 2**A–D), tract-based analysis characterized abnormalities of FA and AD along the AF in schizophrenia. Compared with healthy controls, patients with schizophrenia showed significant reduction in FA (**Figure 2**A) along a specific region of the left AF which is close to the primary motor cortex (BA4) and the supplementary motor area (BA6) (see **Figure 2**E). The same pattern of changes was mirrored on the right side but to a lesser degree without reaching the level of significance.

### AF Asymmetry

From ROI-based analysis, LI of FA was greater than 0 in both controls and patients with schizophrenia (controls: t = 5.67, p<0.001; schizophrenia: t = 5.37, p<0.001), suggesting leftward asymmetry of the AF in both groups. However, LI of AD was greater than 0 in controls but not in patients with schizophrenia (controls: t = 4.48, p<0.001; schizophrenia: t = −0.03, p = 0.973). No group difference in LI of FA or AD was found between the two groups.

**Figure 3:**
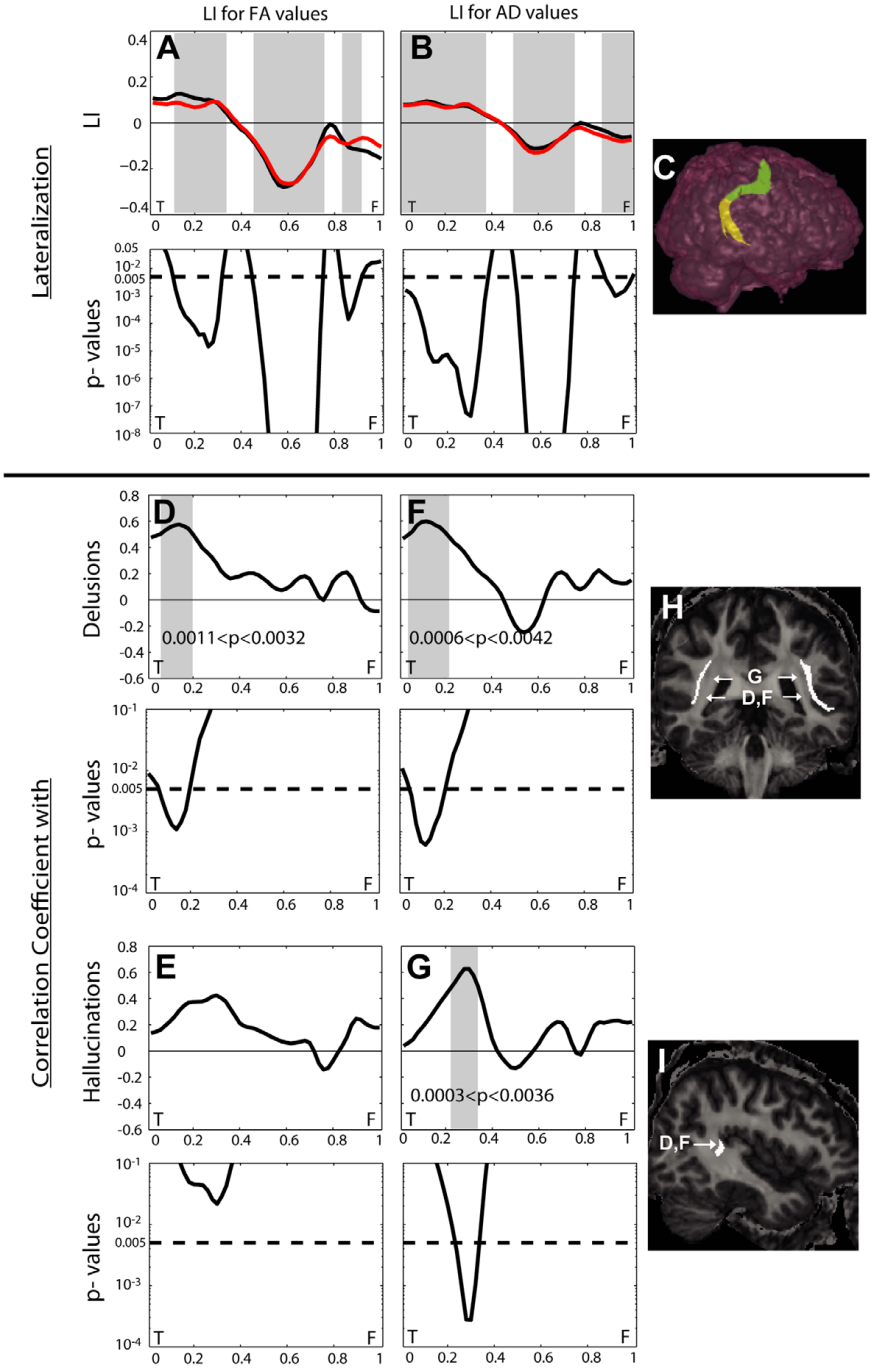
Lateralization index (LI) profiles of the arcuate fasciculus (AF) in fronto (F)-temporal (T) span and their correlations with clinical scores. On the top row of columns (A, B), the black line denotes the LI profile averaged over healthy controls, while the red line denotes the profile averaged over patients with schizophrenia. The regions in gray denotes the anatomical locations with significant lateralization away from zero at p<0.05 after Bonferroni correction as marked by the dashed line on the bottom row of columns (A, B). On panel (C), the AF superimposed on the 3D lateral view of the brain is colored in yellow for left lateralization and in green for right lateralization. On the top row of columns (D–G), the line denotes correlation profile with clinical scores in patients with schizophrenia. The regions in gray denotes the anatomical locations with significant correlations with clinical scores at p<0.05 after Bonferroni correction as marked by the dashed line on the bottow row of columns (D–G). Arrows on panels (H, I) point to the AF locations with significant correlations shown on panels (D, F, G).

Using tract-based analysis, the LI profile along the AF in **Figure 3** [48] shows shaded regions where LI of FA and AD was significantly less than 0 towards the location of the frontal lobe while LI of FA and AD was significantly greater than 0 towards the location of the temporal lobe within controls and patients with schizophrenia. This suggests that there is transition of left lateralization to right lateralization from the temporal lobe to the frontal lobe within the AF, which is illustrated in **Figure 3**C. There were no significant differences in LI of FA or AD between controls and patients with schizophrenia at every location of the AF.

### Correlation of AF indices with psychopathology

No significant correlations of FA and AD values of the AF ROI were found with PANSS total as well as positive, negative and general psychopathology symptom subscale scores after controlling for the effects of age, years of education, and illness duration.

Amongst patients, tract-based analysis found significant positive correlations between LI of FA and LI of AD values in the temporal loci of the AF with the PANSS subscore for delusions (**Figure 3**D, 3F). There were also significant positive correlations of LI of AD values with hallucinations in slightly more medial temporal loci the AF (**Figure 3**G). Even though we observed a trend of correlation patterns of LI of FA with hallucinations (0.022<p<0.044) similar to that LI of AD, such correlations were not significant after Bonferroni correction (**Figure 3**E).

## Discussion

To the best of our knowledge, this is the first study that sought to identify regionally specific abnormality and lateralization of AF white matter indices and their association with positive symptoms of schizophrenia. Patients with schizophrenia had lower FA in the frontal aspects of the left AF compared with healthy controls. Beyond global leftward asymmetry of the AF yielded from ROI-based analysis, more detailed tract-based analysis revealed that such global leftward asymmetry varies along the AF tract in both groups. The AF appeared right lateralized in the frontal lobe and left lateralized in the temporal lobe in terms of FA and AD measures. In particular, greater left lateralization at specific loci of the AF in the temporal lobe was associated with more severe positive psychotic symptoms such as delusions and hallucination in patients with schizophrenia.

Earlier reports using different methods have found either an increase or reduction in FA of the AF in schizophrenia [10], [11], [49]. One ROI-based analysis [49] and two voxel-based analysis studies [10], [50] found reductions in FA of the AF in schizophrenia. However, using voxel-based analysis, Rotarska-Jagiela et al. [21] found that the AF was the only tract that showed increased FA in schizophrenia in contrast to decreased FA in other associative fibers including inferior longitudinal fasciculus and the corpus callosum. Hubl et al. [11] employed voxel-based analysis and found increased left FA of the AF only in patients with auditory hallucinations but in patients without auditory hallucinations compared to healthy controls. These discrepancies may be partially due to differences in patient populations or anatomical definition of the AF.

Improving upon earlier studies that mainly focus the AF passing through the temporal lobe to Broca's area, our study employed tract-based analysis and identified the AF also as the most prominent bundle connecting the superior longitudinal fasciculus and the precentral gyrus, which was also noted in recent studies [34], [35], [36]. We found that FA reductions of the AF in schizophrenia occurred in areas corresponding to the left premotor cortex and supplementary motor cortex. The reductions in FA may reflect decreased myelination, loss of axonal membrane or loss of coherence [51], [52] in the frontal AF, suggesting that disruptions of fronto-parieto-temporal connections may occur through these pathways [53], [54]. Furthermore, such abnormalities in the premotor and supplementary motor cortices may disrupt a top-down inhibitory control process necessary for distinguishing the source of hallucinations as inner thought rather than from an external source [55]. This deficit is possibly influencing and affecting auditory and speech production areas during overt and inner speech [15], [16] which can underlie psychotic experiences such as auditory hallucinations.

Our tract-based analysis also revealed AF lateralization patterns in both groups in that there was leftward (left-greater-than-right) FA and AD asymmetry in the temporal segment and rightward (right-greater-than left) FA and AD asymmetry in the frontal segment. This was not observed using ROI-based analysis in this study when the entire AF tract was considered as whole or in previous studies using voxel-based analysis [56]. Dominant leftward asymmetry in the temporal lobe has been found in postmortem and MRI morphological studies [57], [58] particularly in structures within the temporal-parietal cortex such as the planum temporale [58] and Heschl's gyrus [59]. Combined with findings of the rightward AF asymmetry in the frontal lobe, these morphologies may reflect differences in regional neurodevelopment, particularly late developing temporo-parietal cortex [60], [61] involving processes such as neurogenesis, pruning and formation of neural networks [62].

We found that leftward FA asymmetry in the temporal aspects of the AF corresponding to the angular gyrus as well as at the posterior portions of Wernicke's area. This leftward FA asymmetry correlated with severity of positive symptoms in schizophrenia, specifically delusions and hallucinations. This finding is congruent with those found in its adjacent gray matter structures. Structural anomalies of the left temporal lobe, specifically the superior temporal gyrus, have been previously found to be associated with the presence and severity of positive symptoms, particularly hallucinations [28], [63]. Several studies have also linked lateralized anomalies in temporo-parietal volumes in patients with hallucinations to functional measures of auditory laterality [64], [65] but our strong evidence of a link between lateralized white matter anomalies and positive symptoms adds a new dimension to the previously reported associations.

The above finding can be interpreted from a neurodevelopmental perspective. In normal development, the AF demonstrates marked morphological changes during childhood and adolescence [66] that may be the result of a programmed imbalance in the distribution of AF axons that leads to hemispheric specialization [67]. Postmortem work indicates that axons of the left posterior superior temporal lobe are more thickly myelinated than those in the right, suggesting a possible explanation for left-hemispheric dominance, rapid sensory signal processing, and functional asymmetry for language [57]. Animal and in vivo tractographic studies show that the AF connects language related regions in the frontal, dorsolateral parietal and temporal areas [68] and forms a network of parieto-temporal-frontal connections that participate in language processing as well as working memory, aspects which are disrupted in schizophrenia [17].

The greater leftward lateralization of AD and FA in the temporal AF which correlates with positive psychotic symptoms may be explained by a persistent and relative deficiency in the number of axonal bundles within the right AF compared to the left in schizophrenia [67]. Hence, the difference of white matter integrity of the left frontal AF and accentuation of normal left greater than right asymmetry of FA/AD in the temporal AF suggests aberrant fronto-temporal connectivity. The dysconnectivity between frontal and temporal brain regions can affect corollary discharge of neural signals from frontal speech/motor initiation areas to suppress activity of auditory cortex which may underlie psychotic phenomena such as auditory hallucinations and facilitate elaboration of delusional content [69], [70].

There are several strengths in this study. The tract-based analysis allows characterization of specific changes along the AF from the frontal to temporal lobes, which is not identified using ROI analyses of the whole AF or voxel-based analyses. The AF extraction is replicable based on Mori's DTI atlas and the delineation protocol as described above. Despite the relatively modest sample akin to that of previous studies, we noted reductions in FA at the frontal segment of AF and correlations of specific asymmetries of the AF with psychopathology. Replication in a larger cohort and at different stages of the illness would allow better understanding of the longitudinal changes of the AF and in relationship with the illness progress and treatment factors.

In conclusion, we found differential abnormalities in the white matter integrity of the AF in terms of reduced FA in the frontal segment and that accentuation of left greater than right asymmetry of FA/AD in the temporal segment correlated with positive psychotic symptoms in schizophrenia. Because of the importance of the AF in fronto-parietal-temporal connectivity and impact on language and working memory functions which are affected in schizophrenia, further delineation of specific changes and their relationship with clinical, neurocognitive parameters and longitudinal changes are needed with the potential to better understand the underlying neural basis of this condition.
